# Variability and change over time of weight and BMI among adolescents and adults with Prader-Willi syndrome: a 6-month text-based observational study

**DOI:** 10.1186/s13023-020-01504-7

**Published:** 2020-09-03

**Authors:** Caroline J. Vrana-Diaz, Priya Balasubramanian, Nathalie Kayadjanian, Jessica Bohonowych, Theresa V. Strong

**Affiliations:** 1 Foundation for Prader-Willi Research, 340 S. Lemon Ave, #3620, Walnut, CA 91789 USA; 2 Present Address: Citizens United for Research in Epilepsy, Chicago, USA

**Keywords:** Prader-Willi syndrome, Obesity, Hyperphagia, Technology

## Abstract

**Background:**

Prader-Willi syndrome (PWS) is a rare neurodevelopmental disorder in which hyperphagia (excessive appetite) is a hallmark feature. Understanding how weight changes over time in this population is important for capturing the contemporary natural history of the disorder as well as assessing the impact of new treatments for hyperphagia. Therefore, we aimed to determine the feasibility of a remote assessment of weight change over time in PWS.

**Methods:**

We developed a text message-based, prospective cohort study of adolescents and adults with PWS to assess changes in weight and body mass index (BMI) over a six-month period. Weight was collected weekly, while changes in height, living situation, access to food, activity level, and medication were collected at three-month intervals.

**Results:**

One hundred and sixty-five participants enrolled in the study, with a mean age of 19.7 years (range 12–48). There was considerable variability in weight across participants (range: 76.8–207.7 kg). Thirty-three percent of the participants were normal weight, while 15% were overweight and 52% were obese. Overall, the weight of the study participants increased over the study period (mean weight change + 2.35%), while BMI was relatively stable, albeit high (mean BMI of 31.4 at baseline, mean BMI percent change + 1.42%). Changes in living situation, activity, food access, and medication had limited impact on weight and BMI changes. Multivariable analysis found that time, sex, age, and percentage of life on growth hormone (GH) therapy were statistically significant fixed effects. Participants submitted more than 95% of possible weight data points across the 26 weeks of the study.

**Conclusions:**

This remote, observational study of weight change in PWS showed small increases in weight and BMI over a six-month period. Participants were highly compliant with this text message-based study, suggesting that mobile technology-based data collection was manageable for the participants. We anticipate that the results of this study will inform clinical trials for hyperphagia/obesity related therapies in PWS and provide a basis for understanding the efficacy of new therapies for hyperphagia in the real-world setting.

## Introduction

Prader-Willi syndrome (PWS) is a complex genetic disorder caused by the loss of expression of paternally inherited, imprinted genes on chromosome 15q11.2-q13.1 [1, 2]. The estimated birth prevalence of PWS is approximately 1 in 15,000 individuals, similar in both sexes, and present in all races and ethnic groups [2]. PWS is a multisystem disorder that includes endocrine dysfunction, developmental delay, hyperphagia, obesity, and behavioral problems as individuals age [3–5].

The initial clinical course of PWS is characterized by hypotonia in infants, with decreased movement, lethargy, feeding difficulties, and failure to thrive [6]. A defining feature of PWS is the change in appetite over time, with the onset of hyperphagia, an intense, unrelenting sensation of hunger, sometime after early childhood [6]. Adolescents and adults with PWS will become morbidly obese if strict environmental controls limiting food intake are not implemented. In addition to increased appetite and decreased satiety [6], the propensity for obesity is exacerbated by decreased physical activity, altered body composition and a reduced metabolic rate [7]. Obesity is associated with a number of complications, such as cardiovascular disease, diabetes mellitus, hypertension, sleep disturbances (including sleep apnea), poor quality of life, and increased mortality in PWS [8–10]. Despite improvements in early diagnosis of PWS, all-cause mortality of those with PWS remains significantly increased compared to the general population [11, 12].

Recombinant growth hormone (GH) therapy was approved for children with PWS in the United States in 2000 [13], and remains the only approved medication for PWS. GH is effective in normalizing growth and improving body composition in PWS, with additional positive effects on cognition and adaptive behavior, but has no effect on hyperphagia [14–17]. To date, no Food and Drug Administration (FDA)-approved drugs have proven effective in controlling appetite and food-related behavior in PWS, however, several medications are currently undergoing evaluation in clinical trials to assess impact on hyperphagia in PWS [14].

Earlier diagnosis and family education offer an opportunity for improved weight control in children with PWS [18], but there is currently limited information on weight variability and changes in weight over time in adolescents and adults with PWS living in the home setting. Gathering a reference dataset of PWS weight change in the real-world setting is critical for understanding the natural history of the disorder, and for assessing the impact of new drugs in development for PWS. Given the dispersed, rare population, as well as challenges with transporting individuals with PWS to expert clinical sites, we sought to explore the feasibility of an entirely remote assessment of weight change over time in this population. Thus, we developed a text-based observational study with the primary objective of assessing the variability in weight and BMI, as well as change over time in weight and BMI over 6 months in PWS adolescents and adults (12 years and older). Information about changes in environmental factors that could either directly or indirectly impact weight change, such as changes in diet, medication, food access, and the living environment, were also collected.

## Methods

### Study design and population

This study was a six-month text message-based prospective cohort study in PWS adolescents and adults aged 12 years and older. Participants were recruited through PWS patient advocacy groups in both the United States and Canada through social media, the Global PWS Registry [19], and email. When possible, informed consent was given by the individual with PWS. However, when appropriate, informed consent was given by the parent or legally authorized representative (LAR), and the individual with PWS provided assent before enrollment into the study. The only exclusion criterion for the study was age; participants were not excluded based on other co-morbid conditions. The diagnosis of PWS was reported by the parent/LAR and was not independently verified.

### Data collection

A commercially available text messaging-based clinical platform from Mosio (https://www.mosio.com) was used to collect data. Mosio is Health Insurance Portability and Accountability Act (HIPAA) and Code of Federal Regulations (CFR) Part 11 compliant. This study was reviewed and approved by an independent institutional review board (Hummingbird IRB). For data collection, the majority of text messages were provided by the parent/LAR of the individual with PWS, while a small number of participants with PWS provided data directly for themselves.

### Exposures

**Fig. 1:**
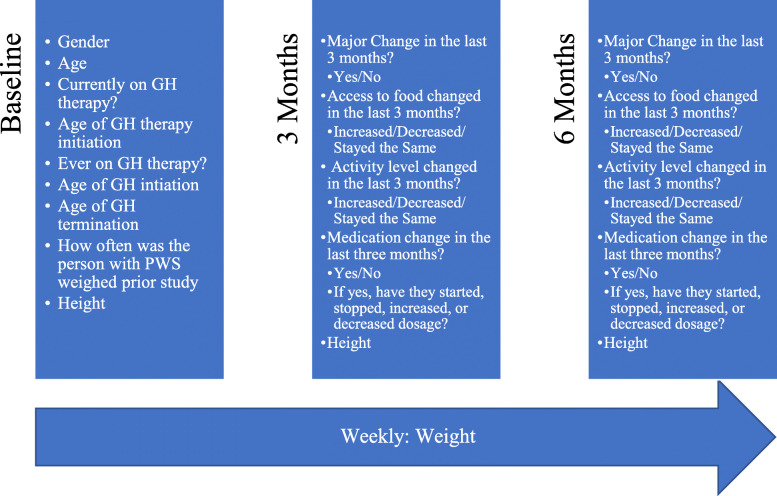
Flow chart of information requested through text-based messaging during the six-month study period. Questions were asked at baseline, three months post-baseline, and six-months post baseline, while weight was assessed weekly throughout the study period

The flow chart of questions asked throughout the study can be seen in Fig. 1. Sex, age, and growth hormone (GH) therapy questions (whether they are currently on therapy, were ever on therapy, and length of use) were collected at baseline. A composite variable for percentage of life on GH therapy was created by dividing the number of years on GH therapy (calculated by the ages of starting and stopping GH therapy if currently not on therapy, or the difference between the current age and the age at initiation for those currently on therapy) by age. Changes in the last 3 months (living situation, access to food, activity level, and medication changes) were collected at three-month intervals.

### Outcomes

The outcomes for this study were weight, BMI, and percent change in weight and BMI. Weight was collected once a week, every week, for 6 months by text message reminder and reply at the same time every week (7 am every Tuesday morning). If data was entered for weight within 3 days of the original date, it was considered part of that week. BMI was calculated at three specific time points (enrollment, 3 months, and 6 months). Height data for BMI was collected at enrollment, 3 months, and 6 months, and weight data from up to 2 weeks earlier for those time points was used due to missing measurements. Only those participants who had at least 50% of the weight measurements were included in the outcomes of weight, BMI, percent change in weight, and percent change in BMI. BMI categories for adolescents 20 years and younger were calculated using the gender-specific body mass index-for-age percentiles from the US Centers for Disease Control and Prevention (CDC) [20, 21]. BMI categories for adults over 20 years were calculated from the US CDC adult BMI weight status categories [22]. Percent change in body weight and percent change in BMI were measured by the difference between baseline and week 25, for a total of 26 weeks of data (or up to 2 weeks earlier if no weight data was available for week 25).

### Analyses

Results are presented for participants who had at least one post-baseline weight measurement. Both mean plots and individual-level plots over time were created. Cochran Mantel Haenzel tests and two sample t-tests were used for demographic bivariate analysis. For the outcomes of weight and BMI, repeated measures analysis of variance (ANOVA) and Cochran Mantel Haenzel tests were used for bivariate analysis, and generalized estimating equations were used for population-specific multivariable analysis. Quasi-information criteria (QIC) was used to determine the best model for multivariable analysis, as well as individual significance of variables (*p*-value < 0.05 was used for significance). A log-normal distribution was used for both weight and BMI outcomes due to the study population’s distribution. For the outcomes of percent change in weight and BMI, analysis of variance (ANOVA) was used for bivariate analysis, and generalized linear models were used for population-specific multivariable analysis. R^2^ was used to determine the best model, as well as individual significance of variables (*p*-value < 0.05 was used for significance). SAS 9.4 (SAS Institute, Inc., Cary, NC) was used for all analysis.

## Results

Table 1 shows the demographics for this study population at enrollment. Overall, 165 participants enrolled in the study (50.9% male). The mean age of the population was 19.7 years (SD 7.9 years), and ages ranged from 12 to 48 years. The overall mean height at enrollment was 157.2 cm (SD 13.2 cm), the mean height for men at enrollment was 163.2 cm (SD 12.8 cm) and the mean height for women at enrollment was 151.3 cm (SD 10.7 cm). At enrollment, the mean weight of all participants was 76.8 kg (SD 30.7 kg), with a median of 72.3 kg, but there was very high variability (weights ranged from 33.2 kg to 207.7 kg). The mean BMI at enrollment was 31.4 kg/m^2^, median BMI of 28.6 kg/m^2^, also with high variability (BMI ranged from 13.2 kg/m^2^ to 88.0 kg/m^2^). At enrollment, the BMI categories were 0.6% underweight (1/161), 32.3% normal weight (52/161), 14.9% overweight (24/161), and 52.2% obese (84/161), and did not differ for under 18 compared to 18+ age groups (Table 2). The overall distribution of BMI categories did not significantly change over time (*p*-value 0.95), so the majority of this study population was obese at every time point (52.2% at baseline, 53.5% at 3 months, and 52.9% at 6 months).

**Table 1:** Characteristics of 165 study participants with Prader-Willi syndrome in a remote six-month text-based study

Characteristics	Study Participants (***n*** = 165) n (%)
Sex (Male)	84 (50.9)
Age at enrollment (years), mean + SD	19.7 + 7.9
Age range (years)	12–48
Age categories	
12–17	88 (53.3)
18+	77 (46.7)
Height at Enrollment (cm), mean + SD	157.2 + 13.2
*Missing*	12 (7.3)
Weight at Enrollment (kg), mean + SD	76.8 + 30.7
Weight at Enrollment (kg), median	72.3
*Missing*	2 (1.2)
Weight Range at Enrollment (kg)	33.2–207.7
BMI at enrollment, mean + SD	31.4 + 12.7
BMI at enrollment, median	28.6
*Missing*	4 (2.4)
BMI Range at Enrollment	13.2–88.0

*SD* Standard deviation**,** *cm* Centimeters, *kg* Kilograms

**Table 2:** BMI category distribution at enrollment among 165 study participants with Prader-Willi syndrome

BMI-for-age categories	12–17 years old, n (%)	18 years or older, n (%)	Total
Underweight	1 (1.2)	0 (0)	1 (0.6)
Normal Weight	29 (33.3)	23 (31.1)	52 (32.3)
Overweight	10 (11.5)	14 (18.9)	24 (14.9)
Obese	47 (54.0)	37 (50.0)	84 (52.2)
*Missing*			4 (2.4)

BMI categories for adolescents 20 years and younger were calculated using the gender-specific body mass index-for-age percentiles from the US CDC. Adult BMI categories were assigned from the US CDC categories

Table 3 shows the growth hormone therapy use in the study population. Almost 60% (98/165) of the population was currently on GH therapy during the study. Those younger than 18 were significantly more likely to currently be on GH therapy (78.4% versus 37.7%, *p* < 0.0001) and ever have been on GH therapy (93.2% versus 72.7%, *p* = 0.0004) compared to those who are 18 or older. The mean number of years that the study population had been on GH therapy was 9.51 (SD 6.48), and the mean percentage of life on GH therapy was 56.7% (SD 37.7%). While the number of years on GH therapy was not statistically different between age groups, those younger than 18 years had a statistically significantly higher percentage of their life on GH therapy (70.5% vs. 40.8%) compared to those 18 years or older (*p* < 0.0001).

**Table 3:** Growth hormone therapy use among 165 study participants with Prader-Willi syndrome

	12–17 years old, n (%)	18 years or older, n (%)	Total
Currently on Growth Hormone*			
Yes	69 (78.4)	29 (37.7)	98 (59.4)
No	19 (21.6)	48 (62.3)	67 (40.6)
Ever on Growth Hormone*			
Yes	82 (93.2)	56 (72.7)	138 (83.6)
No	6 (6.8)	21 (27.3)	27 (16.4)
Number of Years on GH Therapy, mean + SD	9.82 + 4.67	9.15 + 8.11	9.51 + 6.48
Percentage of Life on GH Therapy, mean + SD*	70.5% + 32.9%	40.8% + 36.7%	56.7% + 37.7%

For Current and Ever: Cochran-Mantel Haenzel; For Number and Percentage: two sample t-tests; * *p* < 0.001

**Fig. 2:**
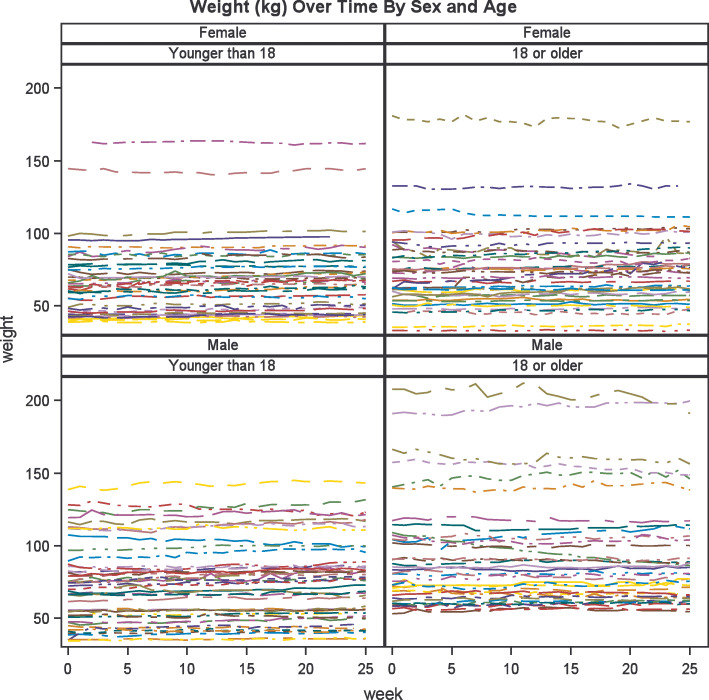
Weight trajectories over the 26 weeks of the study, stratified by age category and sex. Each line represents a single participant. Weight is provided in kilograms. As a group, males 18 and older have higher weights than males younger than 18, while the weight trajectories for females are similar between age categories. Overall, participants had relatively stable weights throughout the six-month study.

**Fig. 3:**
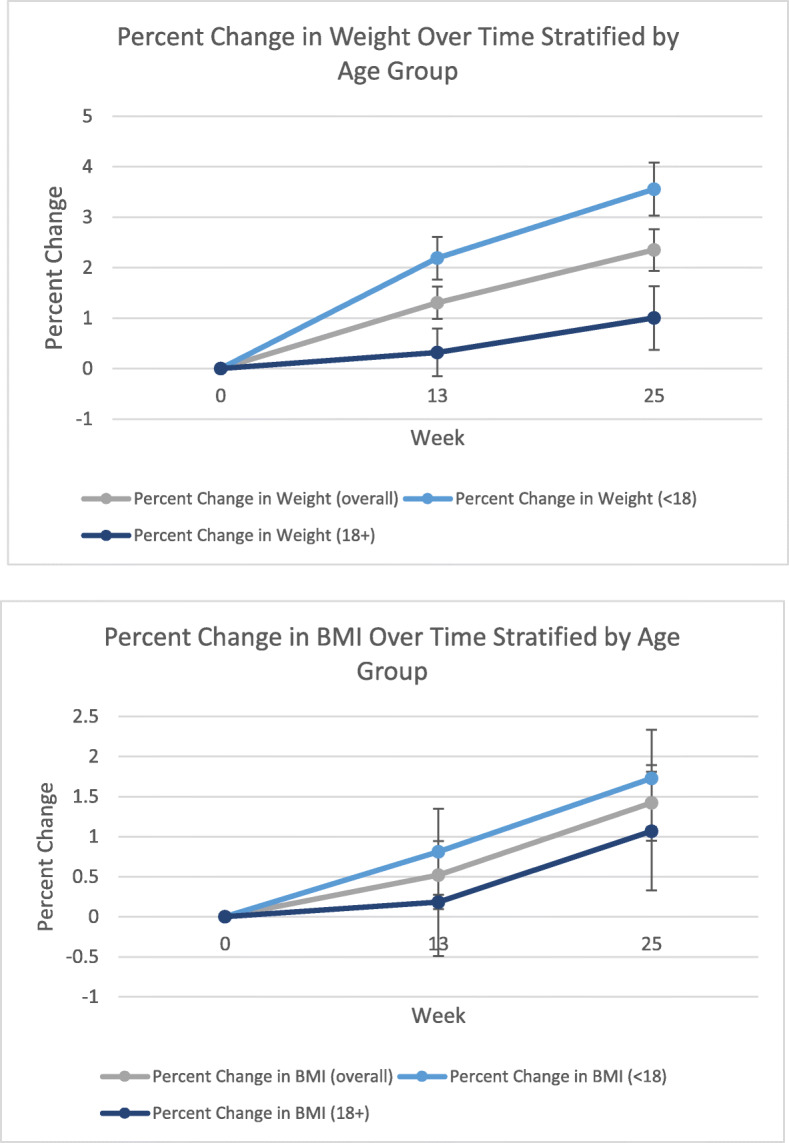
Mean percent weight and BMI change (with standard error bars) measured at three-months post-baseline and six-months post-baseline in 165 study participants with PWS, overall and stratified by age group (< 18 and 18+). The mean percent weight change from baseline to six months post-baseline was + 2.35% (Under 18: + 3.56%; 18+: + 1.00%), and the mean percent BMI change from baseline to six months post-baseline was + 1.42% (Under 18: + 1.73%; 18+: + 1.07%)

**Fig. 4:**
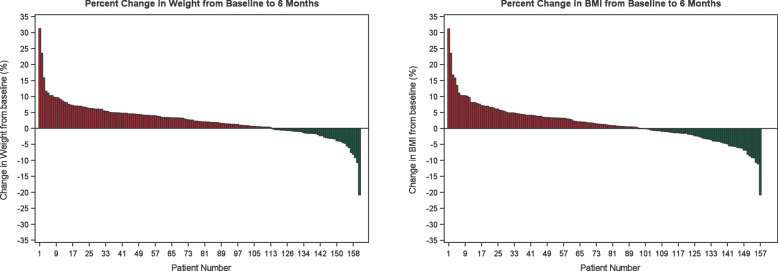
Waterfall plots of the percent change in weight and BMI from baseline to the end of the study. Each bar represents a single participant. Both the percent weight change and percent BMI change in the study population ranged from − 20.8 to + 31.2%. A minority of individuals demonstrated a change in weight and/or BMI of greater than 5%

Overall, the weight of the study participants increased over the 26 weeks of the study, with the mean weight increasing 1.8 kg, from 76.8 kg to 78.6 kg. Figure 2 shows the plots of individual weight change throughout the study, stratified by sex and age. The mean BMI throughout the study was relatively stable, albeit high (mean BMI of 31.4 kg/m^2^, median BMI of 28.6 at baseline, mean BMI of 31.3 kg/m^2^, median BMI of 28.7 at 3 months, and mean BMI of 31.5 kg/m^2^, median BMI of 29.0 at 6 months). The mean percent weight change from baseline to week 25 was + 2.35% (Fig. 3), median + 2.02%. However, the percent weight change in the study population ranged from − 20.8 to + 31.2% (Fig. 4). The percent change in weight for those under 18 was significantly higher than those 18 and older (+ 3.56% versus + 1.00%, *p* = 0.002) (Fig. 3). The percent change in weight by weight status was as follows: + 3.07% for those with underweight or normal weight, + 3.63% for those with overweight, and + 1.66% for those with obesity (data not shown).

The mean percent BMI change from baseline to 6 months was + 1.42% (Fig. 3), median + 0.95%, but the percent BMI change in the study population ranged from − 20.8 to + 31.2% (Fig. 4). The percent change in BMI for those under 18 and those 18 and older did not significantly differ (+ 1.73% versus + 1.07%, *p* = 0.49) (Fig. 3). The range of individual percent change in weight and BMI throughout the study population is depicted in Fig. 4. A minority of individuals demonstrated a change in weight (20.6% of the study population) and BMI (17.6% of the study population) of greater than 5% (Fig. 4).

We next looked at factors that might be influencing weight change during the study period (Supplemental Table 1). Table 4 shows the life changes during the study. Overall, most individuals experienced some type of change during the study, with only 20% reporting no changes. Thirty-three percent had a major life change, 30.1% had a change in food access, 53.4% had a change in their activity level, and 43.2% had a change in their medications.

**Table 4:** Life changes during the study among 165 study participants with Prader-Willi syndrome

Changes throughout the study	Study Participants, n (%)
Major Life Change	
Yes	55 (33.7)
No	108 (66.3)
Change to Food Access	
Yes	49 (30.1)
No	114 (69.9)
Change in Activity Level	
Yes	87 (53.4)
No	76 (46.6)
Change in Medications	
Yes	70 (43.2)
No	92 (56.8)
Experienced at least one of the previous changes	
Yes	130 (79.8)
No	33 (20.2)

We performed bivariate analysis using repeated measures ANOVA for the two main outcomes, weight and BMI, and ANOVA for percent change in weight and BMI to inform our multivariate analysis (Table 5). We found significant main effects of time (BMI), sex (weight), age (weight, BMI, percent change in weight), percentage of life on GH therapy (weight, BMI), major life change (BMI), and change in food access (BMI). Main effects were similar between participants below the age of 18 compared to those 18 and older (not shown).

**Table 5:** Significant bivariate analysis of weight, BMI, and percent change in weight with demographic variables

Variable	F (df)	F	*P*-value
**Weight**			
Week	25,87	1.34	0.163
Sex (ref = Male)	1110	6.87	0.01**
Age	1110	10.49	0.0016**
Percentage of Life on GH Therapy	1109	7.40	0.0076**
Major Life Change	1110	1.03	0.312
Change in Food Access	1110	3.39	0.0683
Change in Activity Level	1110	3.63	0.0592
Change in Medication	1110	0.90	0.344
**BMI**			
Week	2155	3.13	0.046*
Sex (ref = Male)	1155	0.00	0.98
Age	1155	13.2	0.0004**
Percentage of Life on GH Therapy	1154	34.1	< 0.0001**
Major Life Change	1155	3.96	0.0485*
Change in Food Access	1155	7.23	0.0080**
Change in Activity Level	1155	2.87	0.092
Change in Medication	1155	1.58	0.21
**Percent Change in Weight**			
Sex (ref = Male)	1159	0.43	0.511
Age	1159	9.79	0.0021**
Percentage of Life on GH Therapy	1158	1.34	0.249
Major Life Change	1.159	1.47	0.226
Change in Food Access	1.159	0.00	0.95
Change in Activity Level	1159	1.89	0.171
Change in Medication	1158	0.28	0.598
**Percent Change in BMI**			
Sex (ref = Male)	1155	2.23	0.137
Age	1155	0.12	0.727
Percentage of Life on GH Therapy	1154	0.32	0.575
Major Life Change	1155	0.32	0.572
Change in Food Access	1155	0.01	0.932
Change in Activity Level	1155	0.00	0.951
Change in Medication	1155	0.35	0.557
**Weight**			
Week	25,87	1.34	0.163
Sex (ref = Male)	1110	6.87	0.01**
Age	1110	10.49	0.0016**
Percentage of Life on GH Therapy	1109	7.40	0.0076**
Major Life Change	1110	1.03	0.312
Change in Food Access	1110	3.39	0.0683
Change in Activity Level	1110	3.63	0.0592
Change in Medication	1110	0.90	0.344
**BMI**			
Week	2155	3.13	0.046*
Sex (ref = Male)	1155	0.00	0.98
Age	1155	13.2	0.0004**
Percentage of Life on GH Therapy	1154	34.1	< 0.0001**
Major Life Change	1155	3.96	0.0485*
Change in Food Access	1155	7.23	0.0080**
Change in Activity Level	1155	2.87	0.092
Change in Medication	1155	1.58	0.21
**Percent Change in Weight**			
Sex (ref = Male)	1159	0.43	0.511
Age	1159	9.79	0.0021**
Percentage of Life on GH Therapy	1158	1.34	0.249
Major Life Change	1.159	1.47	0.226
Change in Food Access	1.159	0.00	0.95
Change in Activity Level	1159	1.89	0.171
Change in Medication	1158	0.28	0.598
**Percent Change in BMI**			
Sex (ref = Male)	1155	2.23	0.137
Age	1155	0.12	0.727
Percentage of Life on GH Therapy	1154	0.32	0.575
Major Life Change	1155	0.32	0.572
Change in Food Access	1155	0.01	0.932
Change in Activity Level	1155	0.00	0.951
Change in Medication	1155	0.35	0.557

For weight, 26 weekly weight measurements were used. For BMI, 3 time points were used (baseline, 3 months, 6 months). For weight/BMI: repeated measures ANOVA used, for percent change in weight/BMI: ANOVA; *p < 0.05, **p < 0.01

Table 6 shows the multivariable modeling for weight over time and BMI over time. For the outcome of weight, time in the study, sex, and percentage of life on GH therapy were statistically significant fixed effects. For every week increase in the study, participants’ weight significantly increased by 0.06% (95% CI 0.02–0.09%). This means that at the end of the six-month study, weight had increased by 1.56%. Overall, weight for females in this study was 15% less than males (95% CI 4.4–24.7%). For every percentage of life on GH therapy increase, weight was decreased by 0.22% (95% CI 0.1–0.4%). For BMI, percentage of life on GH therapy was a significant fixed effect. For every percent of life on GH therapy increase, BMI was decreased by 0.41% (95% CI 0.2–0.6%). For the multivariable modeling of percent change in weight, we found that age was a significant fixed effect. For every year increase in age, percent change in weight decreased by 0.20% (SE 0.07), *p* = 0.0029. There were no significant fixed effects for percent change in BMI.

**Table 6:** Multivariate modeling for weight and BMI among 165 study participants with Prader-Willi syndrome

Variable	Exponentiated Beta (95% CI)	Type 3 GEE Analysis Chi-Square	*P*-value
**Weight**			
Week	1.0006 (1.0002, 1.0009)	8.48	0.0036**
Sex (ref = Male)	0.850 (0.753, 0.956)	4.83	0.028*
Percentage of life on GH Therapy	0.9978 (0.996, 0.999)	4.46	0.035*
Age	1.006 (0.996, 1.017)	0.06	0.80
**BMI**			
Week	1.0003 (0.9999, 1.0007)	1.34	0.18
Percent of Life on GH Therapy	0.9959 (0.994, 0.998)	14.05	0.0002**
Age	1.0016 (0.994, 1.009)	0.09	0.77

For weight, 26 weekly weight measurements were used. For BMI, 3 time points were used (baseline, 3 months, 6 months). Generalized Estimating Equations used; *p < 0.05, **p < 0.01

We had very high compliance among participants in this study. The mean percentage of weeks with a completed weight measurement was 95.1%, with no significant difference by age (96.8% for those 18 and older, 93.6% for those younger than 18). Only 2 people returned less than 50% of their weight measurements, and 68% of the study population returned 100% of their weight measurements for all 26 weeks in the study.

## Discussion

This study is the first of its kind to investigate the real-world variability at baseline and change over 6 months of weight and BMI in a large group of PWS adolescents and adults. Overall, we found that weight and BMI increased over time in this population, with weight increasing by 2.35% (SD 5.3) from baseline to the end of the six-month study, and BMI increasing by 1.42% (SD 5.9)SE 0.47). However, there was considerable variability in this study population, both in the initial weight (from 33.2 kg to 207.7 kg) and BMI measurements (from 13.2 kg/m^2^ to 88.0 kg/m^2^), as well as the change in weight and BMI over time (both ranged from − 20.8 to + 31.2%). While most participants had relatively stable measurements, approximately 20% of individuals demonstrated a change in weight or BMI of greater than 5% over the study period (Fig. 4).

The average weight gained per year in the United States among adults in the general population has been reported as 0.5 kg to 1 kg in one study [23]. Another study reported an average increase of 0.375 kg per year in a 4 year period [24], whereas National Health and Nutrition Examination Survey (NHANES) data showed a 3.9 kg increase over 16 years among adult men (0.24 kg/yr) and 3.1 kg increase over 16 years among adult women (0.19 kg/yr) [25]. Therefore, as the mean weight increase of 1.00% would translate to an increase of 0.84 kg over 6 months in someone with a weight of 84.2 kg (our mean weight at enrollment among participants 18 and older), adults in this PWS population show faster increases in weight compared to the adult United States average.

Data about weight changes is limited in the PWS population, but the current study population showed more stable weight from baseline to week 26 (+ 2.35%) compared to the change from baseline to week 26 in the placebo arm of a recent PWS clinical trial evaluating a drug for hyperphagia and obesity (+ 4.2%) [26]. The current study also had lower standard error in our weight change (SE of 0.42%) compared to that same trial (SE of 0.9%) [26]. This may reflect a selection bias of individuals enrolling in a clinical trial evaluating a drug for hyperphagia, but additional studies will be needed to determine the differences in the study populations (for example, hyperphagia score) [27].

Overall, the percentage of individuals in this study population with obesity was over 50% at all time points (52.2% at baseline, 53.5% at 3 months, and 52.9% at 6 months). This is relatively comparable to the 58.2% of 292 adults with PWS with obesity from the United States in a cross-cultural comparison of PWS populations, as well as 56% of the 102 adults with PWS from the Dutch Prader-Willi Parent Association [28, 29], but is much higher than the obesity rates of the general US population. According to the Centers for Disease Control and Prevention (2015–2016), the percentage of adults aged 20 and over in the general population with obesity was 39.8%, and the percent of adolescents aged 12–19 years with obesity was 20.6% [30]. Our PWS adolescent population also had a much higher rate of obesity compared to a national survey of weight status among children aged 10–17 (2017–2018 Child and Adolescent Health Measure Initiative) and compared to a nationally representative study of US adolescents aged 12–19 years from 2013 to 2014 NHANES surveys (CAMHI rate of obesity: 15.3%, NHANES rate of obesity: 20.6%, our rate of obesity among adolescents under 18: 54%) [31, 32].

The average BMI in our study across all time points was 31.3 kg/m^2^ (SD 12.41). This is higher than the average BMI among 19 adults with PWS living in Oklahoma (mean BMI for males 27.4, SEM 1.58 and for females 25.7, SEM 2.19) [33], similar to a group of 20 people with PWS aged 16–42 living in Norway (BMI 30.9, SD 6.1) [34] and a group of 14 people with PWS in the United States (mean age: 24.3 years, SD 11.3) participating in a food reward circuitry study (BMI 32.1, SD 7.8) [35], and lower than a study of 73 adults with PWS in France (deletion subtype BMI 40.9 + 11.5, UPD subtype 34.6 + 9.6) [36], as well as those enrolled in a pilot study of Exenatide (baseline BMI 41.7, range 34.1–55.0, age range 14.7–24.6 years) [37]. The current study has a large study population given the low population prevalence of PWS, with 165 individuals providing at least one weekly weight. Because this study was entirely remote, with no clinic visit necessary, it may have allowed participation of a broader population of participants than those participating in clinical trials, which would create a more generalizable and representative set of results.

As expected, males had a statistically significantly higher weight than females. However, sex was no longer significant with the outcome of BMI, percent change in weight, or percent change in BMI. Age was significantly associated with percent change in weight, as age increases, the percent change in weight decreases. The percent change in weight for those under 18 was + 3.56%, compared to a percent change in weight of + 1.00% for those 18 and older, which may reflect the fact that some participants in the younger population are likely to still be growing.

This study population was on GH therapy on average 56.7% of their lives (SD 37.7%). The average number of years on GH therapy in the entire population was 9.51 (SD 6.48), and 59.4% of this population was currently on GH therapy. Adolescents younger than 18 in our study population were significantly more likely to currently be on GH therapy, have ever been on GH therapy, and have a higher percentage of their life on GH therapy compared to those 18 or older. Currently, the consensus recommendations for GH therapy initiation is to start before the onset of obesity, which can often start before 2 years of age, as the greatest benefits are shown when treatment is initiated early in life [17, 38]. In this study, increasing percentages of life on GH therapy was associated with both decreased weight and decreased BMI. This is consistent with the findings of other studies, where GH therapy was shown to reduce BMI in 141 children and adults with PWS in France [28] as well as children with PWS in the Netherlands [39]. In a study of 14 young PWS patients, cessation of GH therapy statistically increased BMI standard deviation score up to 2 years after stopping GH therapy, therefore long-term use of GH may be helpful to stabilize BMI [40]. However, percentage of life on GH therapy and current use of GH therapy were not associated with percent change in weight or percent change in BMI from the beginning to the end of our study.

We queried the participants about life changes, including changes in living situation, activity level, access to food and changes is medication during the course of the six-month study. The goal was to begin to understand how changes in the environment might impact weight changes over time. Overall, the majority of the population had at least one change (major life change, change in food access, change in activity level, or medication change) while participating in the study. There were some significant bivariate analyses with these changes, but they were not detected in the multivariate analysis. While the impact of these life changes on weight may warrant future research, additional information regarding the context and details of these changes are necessary to draw any conclusions, and were limited in the current text-based study. However, the overall acceptance of the remote study design with text-based reporting was high, suggesting that it would be feasible to collect more detailed data in future studies.

The remote study design also allowed the recruitment of a large number of participants in a short amount of time (approximately 2 months), considering the prevalence of the disorder. We had very high compliance rates throughout the study, suggesting that text-based data collection was readily accepted and highly manageable for the participants, with the mean percentage of weeks with a completed weight measurement at 95.1%. Only 2 people returned less than 50% of their weight measurements, and 68% of the study population returned all possible weight measurements.

The strengths of this study include the large number of participants for a study of a rare disease, the high compliance of the text-message based data collection methods, and the relatively long time frame of data collection (6 months). There are a few limitations, however. First, we do not have a confirmed genetic diagnosis of PWS or the specific PWS genetic subtype information. There could be some bias in the weight and BMI measurements due to measurement or reporting inaccuracy (e.g. collecting weight and height at home without a standardized scale, not measuring weight and height consistently). We did not include a group of typically developing individuals to compare to the PWS population. Finally, we only asked about medication therapy, and did not ask about additional types of therapy that the participants were receiving.

## Conclusions

Mobile technologies offer new opportunities to understand the natural history of rare disease populations. For PWS, weight and BMI seem to be feasible to measure remotely as a text message-based study. This method of data collection could be very useful for additional studies in the PWS population. We anticipate this study will inform future clinical trials for hyperphagia and obesity-related therapies and provide a basis for understanding how well potential therapies are working in the real-world setting.

## Supplementary information

**Additional file 1: Table S1.** Life change survey questions asking in the remote six month text-message based study.

## Data Availability

The datasets used and analyzed during the current study are available from the corresponding author on reasonable request.
