# Supplementary material for: Variability and change over time of weight and BMI among adolescents and adults with Prader-Willi syndrome: a 6-month text-based observational study
Source: Orphanet J Rare Dis. 2020 Sep 3;15:233. doi: 10.1186/s13023-020-01504-7 (PMC7469274; doi:10.1186/s13023-020-01504-7)
Supplement: Supplementary file 1 — Additional file 1: Table S1. Life change survey questions asking in the remote six month text-message based study. [file 13023_2020_1504_MOESM1_ESM.docx]

**Supplemental Table 1: Life change survey questions asking in the remote six month text-message based study**

| **Question** | **Available Answers** |
| --- | --- |
| 1. Has there been a major change for the PWS participant in the last 3 months (moved, caregiver change, illness, etc.)? | Yes  No |
|  |  |
| 2. For the person with PWS, has access to food changed in the last 3 months? | Increased  Decreased  Stayed the Same |
|  |  |
| 3. For the person with PWS, has the activity level changed in the last 3 months? | Increased  Decreased  Stayed the Same |
|  |  |
| 4. For the person with PWS, has there been medication change in the last 3 months? | Yes  No |
|  |  |
| 5. Have they: | Started new medication  Stopped medication  Increased medication dosage  Decreased medication dosage |
